# SURGICAL TREATMENT OF TIBIAL STRESS FRACTURE: NAIL X PLATE

**DOI:** 10.1590/1413-785220253305e290433

**Published:** 2025-09-22

**Authors:** Paulo Henrique Schmidt Lara, Guilherme Leme Cabello, Danilo Cabral Domingues, Daniel Costa Bezerra, Antônio Bezerra de Albuquerque, Paulo Santoro Belangero

**Affiliations:** 1 Universidade Federal de Sao Paulo (UNIFESP), Faculdade de Medicina Paulista, Centro de Traumatologia Esportiva, Sao Paulo, SP, Brazil.

**Keywords:** Tibia, Stress Fracture, Fracture Fixation, Intramedullary, Tíbia, Fratura por Estresse, Fixação Intramedular de Fraturas

## Abstract

To analyze and compare two surgical treatment methods for tibial stress fractures: intramedullary nailing and tension plate, taking into account functional outcomes and postoperative results. This systematic review was conducted and written in accordance with the guidelines for systematic reviews – PRISMA (Preferred Reporting Items for Systematic Reviews and Meta-Analyses). The bibliographic search was carried out between January and September 2024 in journals indexed in the PubMed, Lilacs, Cochrane and Embase databases. Ten studies were selected. Among them, five were case reports, four were case series, and one was a retrospective cohort study. Intramedullary nailing offers good stabilization and a gradual return to sports activities, but it can cause complications such as knee pain and additional fractures. The anterior tension plate, on the other hand, provides effective stabilization with less anterior knee pain and faster healing times, but it is more invasive and may require implant removal and intensive rehabilitation. The choice between intramedullary nailing and tension plate should be based on a detailed assessment of the individual characteristics of the patient, the nature of the fracture, and the potential risks associated with each technique. ***Level of Evidence IIA; Systematic Review of Cohort Studies.***

## RESUMO

Analisar e comparar dois métodos de tratamento cirúrgico para fratura por estresse da tíbia: haste intramedular e placa de tensão, levando em consideração os desfechos funcionais e resultados pós-operatórios. A presente revisão sistemática foi conduzida e redigida de acordo com as diretrizes para revisões sistemáticas – PRISMA (Preferred Reporting Items for Systematic Reviews and Meta-Analyses). A pesquisa bibliográfica foi realizada entre janeiro e setembro de 2024 nos periódicos indexados nas bases de dados PubMed, Lilacs, Cochrane e Embase. Dez estudos foram escolhidos. Dentre eles cinco relatos de caso, quatro séries de casos, e um estudo coorte retrospectivo. A haste intramedular oferece boa estabilização e um retorno gradual às atividades esportivas, porém pode causar complicações como dor no joelho e fraturas adicionais. A placa de tensão anterior, por sua vez, proporciona uma estabilização eficaz com menor dor anterior no joelho e tempos de consolidação mais rápidos, mas é mais invasiva e pode exigir a remoção do implante e reabilitação intensiva. A escolha entre a haste intramedular e a placa de tensão deve ser baseada em uma avaliação detalhada das características individuais do paciente, da natureza da fratura e dos riscos potenciais associados a cada técnica. ***Nível de evidência IIA; Estudo de Revisão Sistemática de Estudos Coorte.***

## INTRODUCTION

Stress fractures of the tibia, particularly in the anterior region, are common among athletes and military personnel, with a notable prevalence in high-impact activities such as running and jumping.^1,2^ These fractures, often described as the "feared black line" on radiographs, are challenging due to their location on the tension line of the bone, which hinders nonoperative healing.^3,4^ The conservative approach, which includes rest and activity modification, is generally effective for posteromedial cortex fractures but may be inadequate for anterior cortex fractures, which often result in nonunion or prolonged healing.^5^ When nonoperative treatments fail, surgical intervention becomes an option, with two main techniques to consider: intramedullary nailing and anterior tension plate fixation. Intramedullary nails have been successfully used in several cases, providing symptom relief and allowing for a gradual return to activities.^2^ On the other hand, anterior tension plates have shown theoretical advantages, such as better fracture stabilization.^4^ The choice between these methods depends on several factors, including the patient's profile and the severity of the fracture. This study aims to compare the efficacy and functional outcomes of these two treatment methods for tibial stress fractures, to provide evidence-based guidelines for clinical practice.

## MATERIALS AND METHODS

This review was written in accordance with the PRISMA (Preferred Reporting Items for Systematic Reviews and Meta-Analyses) guidelines. The protocol was published in the PROSPERO registry (CDR42024534684). We conducted a systematic review of the PubMed, Lilacs, Embase, and Cochrane databases, using search terms such as "tibial stress fracture," "intramedullary nailing," "plate fixation," and "athletes" (Table 1). Studies evaluating the results of surgical treatments with intramedullary nails and plates in athletes with tibial stress fractures were included.

**Table 1:** Search syntax used in databases.

Database	Search strategy	Total
Base	(‘fracture fixation, intramedullary' OR ‘intramedullary nailing'/exp OR ‘intramedullary nailing' OR ‘bone plate'/exp OR ‘bone plate') AND (‘tibia'/exp OR ‘tibia') AND (‘stress fracture'/exp OR ‘stress fracture') AND [embase]/lim	149
Lilacs	((tibia) AND (fractures, stress)) AND ((fracture fixation, intramedullary) OR (bone plates) OR (fracture fixation, internal) OR (internal fixators)) AND (db:("LILACS"))	1
Pubmed	((("Tibia"[Mesh] AND "Fractures, Stress"[Mesh]))) AND (("Fracture Fixation, Intramedullary"[Mesh] OR "Bone Plates"[Mesh] OR "Fracture Fixation, Internal"[Mesh] OR "Internal Fixators"[Mesh]))	18
Cochrane	Stress fracture AND Tibia AND (Nail OR Plate OR Fracture Fixation, Internal OR Internal Fixator)ail OR Plate OR Fracture Fixation, Internal OR Internal Fixator)	14

### Data collection

The data extracted from the included studies were the number of patients evaluated, percentage of return to sport, consolidation time, consolidation percentage, functional scores (SF-36, FAOS, Lysholm), complication rate, number of patients operated on with intramedullary nails, number of patients operated on with plates, clinical, and functional outcomes.

### Inclusion and exclusion criteria

The inclusion criteria were: (1) randomized clinical trials, (2) prospective cohort studies, (3) retrospective cohort studies, and (4) case reports.

The exclusion criteria were: (1) literature reviews, (2) studies that did not evaluate the outcomes proposed in our research protocol.

### Data extraction

**Figure 1:**
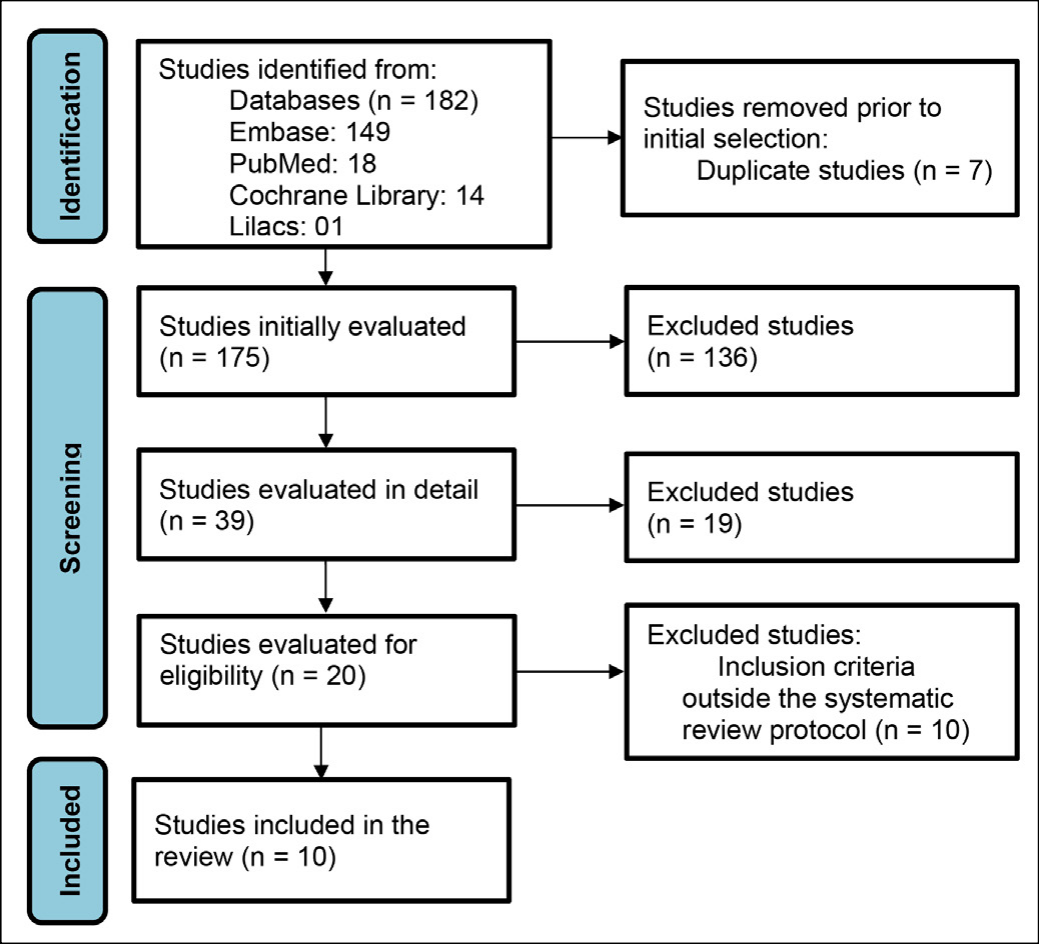
Flowchart of the search for articles in databases.

Two independent researchers reviewed the results to select eligible studies, using pre-established inclusion and exclusion criteria. Disagreements were discussed with a third investigator. (Figure 1)

## RESULTS

Using the search method, 182 potential studies were found in scientific databases, and after applying the exclusion criteria, 10 scientific studies remained. Based on the analysis of the selected articles, it is evident that there is significant diversity in the approaches and results of fracture treatment across different studies. Table 2 summarizes these articles, including the following data: treatment modality used, number of patients, percentage of patients returning to sports, mean time to bone union, percentage of union, and complication rate. Results vary depending on the type of intervention and follow-up provided.

**Table 2:** Evaluation of data and characteristics of the included studies.

Study	Method	Number of Patients	Percentage of Return to Sport	Consolidation Time (weeks)	Consolidation Percentage	Complication Rate	Rod/ Plate	Outcomes	Methodology
Baublitz et al. (2004)^1^	Nail	1	100%	9 months	100%	100%	1/0	New acute fracture. Consolidation and return to sport afterwards.	Case report
Chung et al. (2019)^3^	Nail	1	100%	15 weeks	100%	0%	2/0	Bilateral consolidation and return to sport afterwards.	Case report
Komatsu et al. (2019)^5^	Nail	1	100%	20 weeks	100%	0%	2/0	Bilateral consolidation and return to sport afterwards.	Case report
Martinez et al. (2005)^6^	Nail	1	100%	10 weeks	100%	100%	1/0	Reoperation after refraction. Return to sport after.	Case report
Miyamoto et al. (2009)^7^	Nail	24	100%	6 weeks	100%	0%	3/0	Successful surgical treatment after conservative treatment failure.	Case series
Pandya et al. (2007)^8^	Nail	1	100%	40 weeks	100%	100%	1/0	Reoperation after a new fracture. Return to sport after.	Case report
Cruz et al. (2013)^9^	Plate	3	100%	12 weeks	100%	0%	0/4	Consolidation of all cases. Return to sport after.	Retrospective cohort
Varner et al. (2005)^2^	Nail	7	100%	12 weeks	100%	18%	11/0	Consolidation of all cases. Return to sport after.	Case series
Yamada et al. (2004)^10^	Nail	3	33%	8–12 weeks	100%	100%	3/0	Re-treatment for residual pain. Resolution in 2; 1 in programming	Case series
Zbeda et al. (2015)^4^	Plate	12	92%	9.6 weeks	100%	38%	0/13	High rate of return to sport.	Case series

Studies evaluating the use of rods, such as those conducted by Chung et al.^3^ and Komatsu et al.^5^, have shown a 100% return-to-sport rate, with an average bone consolidation time of 16 weeks and a consolidation rate of 100%. These results suggest that the use of rods may be highly effective in promoting return to sport, especially in patients seeking rapid and complete recovery.

In studies evaluating the use of plates and rods, a slight decrease in the return-to-sport rate was observed, with a rate of 98%, although the average consolidation time was shorter, at 11 weeks. The consolidation rate also remained high at 96%.

Cruz et al.^9^ analyzed the use of plates and rods and reported 100% return-to-sport rates, with a mean consolidation time of approximately 12 weeks and a consolidation rate of 100%.

Studies, such as that by Zbeda et al.,^4^ which involved 13 patients treated with plates, highlighted a 100% consolidation rate and consistent return to sport, despite reporting complications, including medial tibial pain. Medial tibial pain, despite being a significant complication, did not prevent return to sport, suggesting that even with some adversity, treatment with plates may be a viable option.

On the other hand, Miyamoto et al.^7^ reported a longer consolidation time of 20 weeks for patients treated with plates and rods, but still achieved a 100% consolidation rate and a full return to sports. These findings indicate that, although consolidation time may vary, the final efficacy in terms of return to sport and bone consolidation remains high.

Additional studies, such as those by Pandya et al.^8^ and Martinez et al.^6^, which also used rods, demonstrated a 100% return to sport after 40 and 20 weeks, respectively, with no reported complications. These results highlight the durability and effectiveness of the rods in prolonged treatment scenarios, especially when the goal is to ensure a full return to sports.

Yamada et al.^10^ reported a 100% return-to-sport rate within a shorter period of 10 weeks, but with a lower consolidation rate of 33%. This study suggests that although a return to sport can be achieved quickly, complete bone healing may not be guaranteed in all cases, especially when follow-up and treatment are limited.

## DISCUSSION

This systematic review analyzed several studies on the surgical treatment of tibial stress fractures in athletes who used intramedullary nails or tension plates. The reviewed articles provided data on postoperative complications, the percentage of patients returning to sport, fracture healing rates, and healing times.

The main findings indicate that both intramedullary nails and tension plates are viable options for treating tibial stress fractures, but each technique has its advantages and disadvantages. The effectiveness of treatments may vary depending on the type of fracture, the athlete population, and the individual characteristics of the patients.

Complications associated with different surgical techniques were a significant concern. Treatment with intramedullary nailing showed a range of complications, including the possibility of additional acute fractures and persistent pain. Yamada et al.,^10^ for example, discuss complications associated with intramedullary nails in athletes, concluding that there is a high incidence of associated complications and a need for additional surgical intervention. Similarly, Baublitz et al.^1^ report that acute fractures can occur in previously stabilized chronic fractures with intramedullary nails. On the other hand, Varner et al.^2^ in their study of seven athletes treated with intramedullary nails, reported that only one presented with residual pain at the nail insertion point, which was quickly resolved.

Treatment with tension plates also presented complications, although generally less severe. Zbeda et al.^4^ in their study with 12 athletes, reported a 38% need for removal of prominent implants, but without any rate of infection or non-union. The same was reported by Cruz et al.^9^ in their investigation of three high-level athletes treated with tension band using a plate.

The rate of return to sport after surgical treatment was a focal point. The data from the articles indicate that the rate of return to sport is high, but that it can vary depending on the time required to achieve this. Some studies show relatively quick and successful recovery. In contrast, others highlight the need for longer follow-up and intensive rehabilitation to ensure a full return to sports activities, especially when complications such as residual pain and non-union of the fracture are present.

Treatment with intramedullary nails often resulted in healing times ranging from 10 weeks to 40 weeks in a case of nonunion reported by Pandya et al.^8^ On the other hand, tension plate demonstrated potential for faster healing times in studies by Zbeda et al.^4^ and Cruz et al.^9^, however, the exact times may vary depending on the complexity of the fracture and the rehabilitation protocol followed. The studies reviewed showed variations in methodological quality. Some studies presented a solid approach with more details and greater follow-up, while others had limitations due to small sample sizes and less rigorous designs. The lack of standardization in surgical techniques and in the assessment of complications also affected the comparability of results.

The findings suggest that both intramedullary rods and tension plates have distinct advantages and disadvantages. The choice of treatment should consider not only the effectiveness in consolidating the fracture, but also the profile of complications and the rate of return to sport. The decision should be tailored to the individual needs of the athlete and the specific nature of the fracture.

Additionally, implementing strategies to optimize recovery, such as careful monitoring and targeted rehabilitation, is crucial for ensuring a successful return to sport and minimizing complications.

Future research should focus on studies with larger samples and more rigorous designs to directly compare different treatment methods. Longitudinal studies evaluating the effectiveness of rehabilitation approaches and the impact of factors such as nutrition and metabolism on recovery are also needed to provide a more complete understanding.

## CONCLUSION

The results indicate that both approaches are feasible and effective; however, each has its advantages and disadvantages that should be considered when choosing a treatment.

The intramedullary nail has proven to be an effective option, as it stabilizes fractures and allows for a gradual return to sports activities. However, the technique has been associated with a range of complications, such as anterior knee pain and the possibility of additional fractures. On the other hand, the anterior tension plate offers theoretical advantages in terms of fracture stabilization and a lower risk of anterior knee pain, although it is a more invasive approach that may require implant removal and more intensive rehabilitation.
